# A Framework for Understanding the Relationship between Descending Pain Modulation, Motor Corticospinal, and Neuroplasticity Regulation Systems in Chronic Myofascial Pain

**DOI:** 10.3389/fnhum.2016.00308

**Published:** 2016-06-27

**Authors:** Leonardo M. Botelho, Leon Morales-Quezada, Joanna R. Rozisky, Aline P. Brietzke, Iraci L. S. Torres, Alicia Deitos, Felipe Fregni, Wolnei Caumo

**Affiliations:** ^1^Post-Graduate Program in Medical Sciences, School of Medicine, Universidade Federal do Rio Grande do SulPorto Alegre, Brazil; ^2^Pain and Palliative Care Service at Hospital de Clínicas de Porto Alegre, Universidade Federal do Rio Grande do SulPorto Alegre, Brazil; ^3^Laboratory of Pain and Neuromodulation, Hospital de Clínicas de Porto AlegrePorto Alegre, Brazil; ^4^Laboratory of Neuromodulation of Spaulding Rehabilitation of Harvard Medical SchoolBoston, MA, USA; ^5^Pharmacology Department, Institute of Basic Health Sciences, Universidade Federal do Rio Grande do SulPorto Alegre, Brazil; ^6^Department of Physical Medicine and Rehabilitation, Harvard Medical SchoolBoston, MA, USA; ^7^Surgery Department, School of Medicine, Universidade Federal do Rio Grande do SulPorto Alegre, Brazil

**Keywords:** BNDF, cortical excitability, CPM, MEP, TMS, QST, chronic pain

## Abstract

Myofascial pain syndrome (MPS) is a leading cause of chronic musculoskeletal pain. However, its neurobiological mechanisms are not entirely elucidated. Given the complex interaction between the networks involved in pain process, our approach, to providing insights into the neural mechanisms of pain, was to investigate the relationship between neurophysiological, neurochemical and clinical outcomes such as corticospinal excitability. Recent evidence has demonstrated that three neural systems are affected in chronic pain: (i) motor corticospinal system; (ii) internal descending pain modulation system; and (iii) the system regulating neuroplasticity. In this cross-sectional study, we aimed to examine the relationship between these three central systems in patients with chronic MPS of whom do/do not respond to the Conditioned Pain Modulation Task (CPM-task). The CPM-task was to immerse her non-dominant hand in cold water (0−1°C) to produce a heterotopic nociceptive stimulus. Corticospinal excitability was the primary outcome; specifically, the motor evoked potential (MEP) and intracortical facilitation (ICF) as assessed by transcranial magnetic stimulation (TMS). Secondary outcomes were the cortical excitability parameters [current silent period (CSP) and short intracortical inhibition (SICI)], serum brain-derived neurotrophic factor (BDNF), heat pain threshold (HPT), and the disability related to pain (DRP). We included 33 women, (18–65 years old). The MANCOVA model using Bonferroni's Multiple Comparison Test revealed that non-responders (*n* = 10) compared to responders (*n* = 23) presented increased intracortical facilitation (ICF; mean ± *SD*) 1.43 (0.3) vs. 1.11 (0.12), greater motor-evoked potential amplitude (μV) 1.93 (0.54) vs. 1.40 (0.27), as well a higher serum BDNF (pg/Ml) 32.56 (9.95) vs. 25.59 (10.24), (*P* < 0.05 for all). Also, non-responders presented a higher level of DRP and decreased HPT (*P* < 0.05 for all). These findings suggest that the loss of net descending pain inhibition was associated with an increase in ICF, serum BDNF levels, and DRP. We propose a framework to explain the relationship and potential directionality of these factors. In this framework we hypothesize that increased central sensitization leads to a loss of descending pain inhibition that triggers compensatory mechanisms as shown by increased motor cortical excitability.

## Introduction

Myofascial pain syndrome (MPS) is a leading cause of chronic musculoskeletal pain (Simons et al., 1999). MPS has been associated with disability, and also with dysfunction of corticospinal conduction as assessed by motor evoked potential (MEP; Vidor et al., 2014). As with other chronic pain syndromes, the mechanisms of MPS are not entirely elucidated. A major barrier to the understanding of these mechanisms is that pain is an experience orchestrated by a network of cortical regions, elements of the limbic system and the spine-bulbospinal loop. The ascending portion of this circuit involves the spine reticular tract (Willer et al., 1999), which comprises modulatory systems such as the opioidergic (Le Bars et al., 1981; Willer et al., 1990), noradrenergic (Sanada et al., 2009; Makino et al., 2010), and serotonergic systems (Chitour et al., 1982). Given this complex interaction, our approach to provide insights into the neural mechanisms of pain was to investigate the relationship between neurophysiological, neurochemical, and clinical outcomes such as corticospinal excitability as indexed by transcranial magnetic stimulation (TMS) measurements, conditioned pain modulation (CPM) to measure the descendent endogenous inhibitory pain system and serum brain-derived neurotrophic factor (BDNF) as a critical marker of neuroplasticity. Corticospinal excitability as indexed by TMS has become a reliable marker in chronic pain syndromes, including MPS (Vidor et al., 2014). It has been shown that pain and disability are associated with an imbalance between excitatory and inhibitory systems as assessed by increased intracortical facilitation (ICF) and by a reduced current silent period (CSP; Vidor et al., 2014; a proxy of glutamatergic activity), a higher pain catastrophizing score (Volz et al., 2013a) and a higher trait anxiety score (Vidor et al., 2014).

The CPM (Yarnitsky, 2010) involves the diffuse noxious inhibitory control (DNIC) system. The DNIC system assesses the reduction in the pain sensation on the stimulus by a simultaneous pain input from distant sites of the body (Le Bars, 2002). While the CPM assesses how much, a conditioning stimulus can reduce the pain response evoked by the other strong, painful stimuli at a distant large body surface area (the test stimulus; Volz et al., 2013a). When the CPM-task increases pain, this indicates a disruption of endogenous pain-inhibitory processes and a summation effect (King, 2014), which amplifies the pain response and it is a process of the central sensitization (Boyer et al., 2014). It appears that these pain-related neural changes maintain the dysfunction of endogenous descending inhibitory mechanisms as observed in many chronic pain syndromes including knee osteoarthritis (Arendt-Nielsen et al., 2010), chronic pancreatitis (Olesen et al., 2010), rheumatoid arthritis (Leffler et al., 2002a), long-term trapezius myalgia (Leffler et al., 2002b), irritable bowel syndrome (King et al., 2009), temporomandibular disorder (King et al., 2009), fibromyalgia (Staud et al., 2003), and MPS (Pielsticker et al., 2005).

BDNF, a critical molecule for the development and maintenance of cortical neurons and cortical synapses, interacts with the descendant modulatory system. Clinical studies have found higher levels of BDNF in the blood (Deitos et al., 2015) and cerebrospinal fluid in patients with chronic pain (Bø et al., 2009), and in fibromyalgia has been associated with a lower pain threshold (Zanette et al., 2014). This set of evidence demonstrates that there are three main neural systems involved in chronic pain: (i) the corticospinal motor system; (ii) the internal descending pain modulation system; and (iii) the system regulating neuroplasticity. Our hypothesis is that disruption of the infra cortical medulator system, as assessed by pain scores during the CPM-task, is correlated with dysfunction of corticospinal conduction and disinhibition at the cortical level, due to increases in the MEP amplitude, ICF, and serum BDNF level. We aimed to analyze the relationship between these three central systems in chronic MPS patients in responders and non-responders to Quantitative Sensory Testing (QST) during the immersion of her non-dominant hand in cold water (0−1°C) to produce a heterotopic nociceptive stimulus (CPM-task). To determine the CPM we used the difference between the pain score on NPS (0–10) QST during cold water immersion (QST+CPM) at the temperature of the point at which subjects felt 6/10 pain on the NPS scale [during the initial time period (T0)]. Our primary outcomes were the MEP and ICF as assessed by TMS. The secondary outcomes were the cortical excitability parameters [current silent period (CSP) and short intracortical inhibition (SICI)], serum BDNF level, heat pain threshold (HPT), and the disability related to pain.

## Materials and methods

This exploratory study was performed at the Hospital de Clinicas de Porto Alegre in Porto Alegre, Brazil. The study protocol was approved by the Institutional Review Board (IRB 0000921) at the Hospital de Clinicas de Porto Alegre and conducted according to the Declaration of Helsinki. All subjects provided written informed consent for their participation. We administered clinical assessment scales validated in the Brazilian population. Additionally, we collected behavioral measurements (i.e., several pain assessments) and neurophysiological measurements (i.e., motor córtex excitability as indexed by TMS) to establish baseline data.

### Design overview, settings, and participant

We recruited the participants from the general population through public postings in different health care units and physicians' referrals from the Chronic Pain Service at the Hospital de Clínicas de Porto Alegre. The inclusion criteria included the following: (1) right-handed females (2) aged 19–65 years old, (3) confirmed the diagnosis of MPS in the upper body segment for at least 3 months before enrollment, and (4) limitation in routine activities due to MPS. Furthermore, patients needed to present with a pain score of the visual analog scale (VAS) at least of 4 cm (i.e., moderate or severe pain; Palos et al., 2006), associated with functional disability in most days of the 3 months before enrollment. Disability associated with MPS was evaluated using a questionnaire that included six specific questions (yes/no). These questions aimed at assessing interference with work, personal relationships, pleasure obtained during activities, personal goals, clear thinking (i.e., problem solving, concentrating, or remembering), and responsibilities at home during the past 3 months. For enrollment, an affirmative answer to one or more of these questions was necessary to ensure that chronic pain was decreasing the patient's quality of life. Moreover, the diagnosis of MPS was confirmed by a second experienced independent examiner with significant clinical experience related to chronic pain. MPS criteria were the presence of regional pain, normal neurological examination, stiffness in the target muscles; decreased the range of motion, the presence of palpable nodules, tender points, trigger points, taut bands, and pain characterized as hollow, dull, or deep that was exacerbated by stress. To standardize the severity of MPS and to distinguish neuropathic pain from ongoing nociception, were included only patients with the Neuropathic Pain Diagnostic Questionnaire (DN4) with a score equal to or higher than four (Bouhassira et al., 2005). The presence of previous surgery on the affected areas or other pain disorders such as rheumatoid arthritis, radiculopathy, and fibromyalgia; and frequent use of steroidal and non-steroidal anti-inflammatory medications were exclusion criteria.

**Figure 1:**
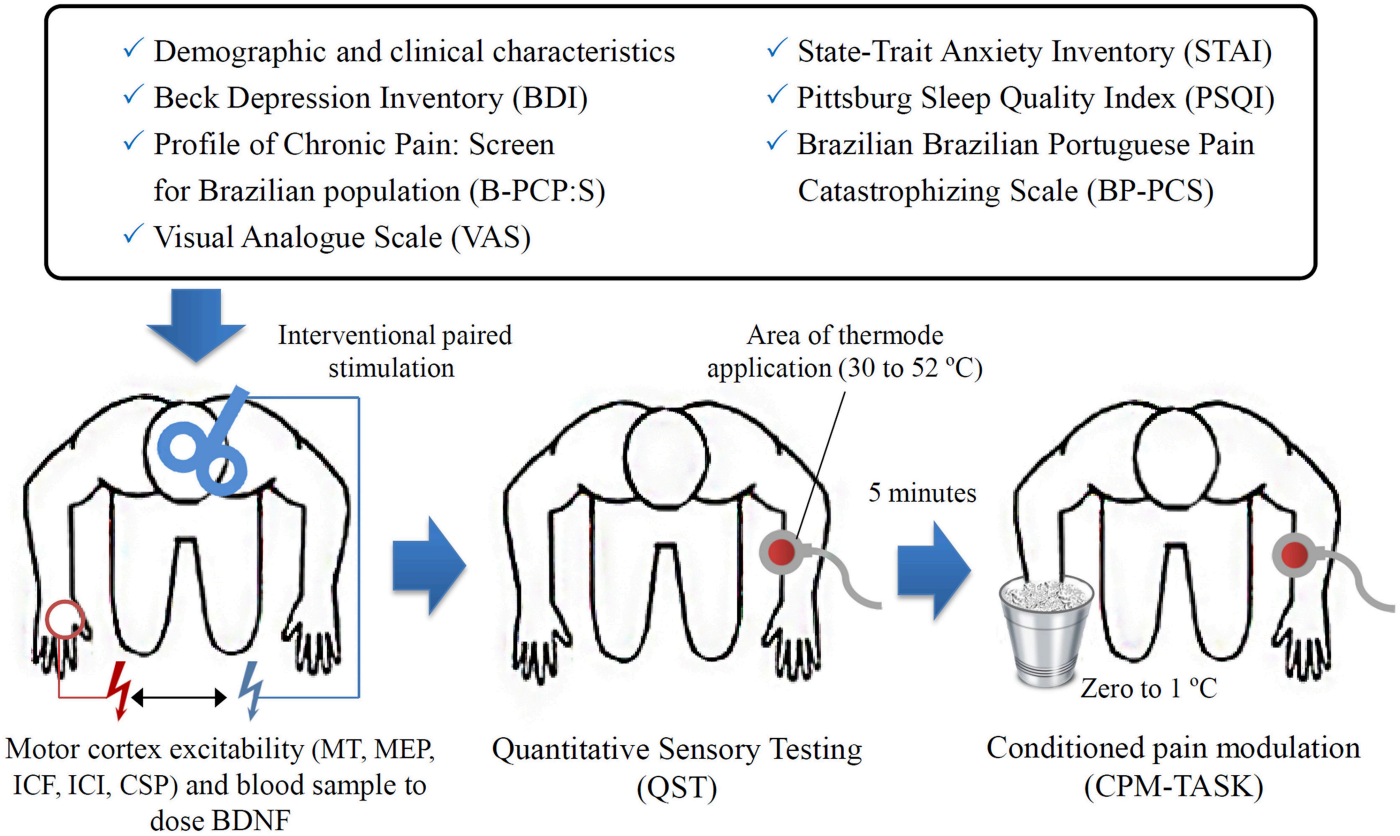
**The sequence of assessments**.

Anticipating an effect size (f) of 0.4 for a multiple regression analysis allowing for two predictors and a type I and II errors of 0.05 and 0.20, respectively, and the minimum sample size was 30 patients. Finally, considering the likely attrition rate and other unexpected factors, the required sample size was determined to be 33 patients (Figure 1).

### Instruments and assessments

The tools used to assess psychological state were validated in the Brazilian population (Staud et al., 2003; Kaipper et al., 2010; Sehn et al., 2012; Caumo et al., 2013). Two independent medical examiners that were blinded to the aim of the study were trained to conduct the psychological tests and to administer the pain scales. The patients' baseline depressive symptoms were assessed using the Beck Depression Inventory (BDI II; Warmenhoven et al., 2012), and the Pittsburgh Sleep Quality Index to assess the sleep quality (Buysse et al., 1989). To measure the anxiety, we used the refined version of the State-Trait Anxiety Inventory (STAI; Kaipper et al., 2010) obtained using the Rasch model, which derivates shorter state-trait STAI-Form X scales free of threshold disorders and for differential item functioning (DIF) problems. The scores in the state- and trait score ranges from 13 to 52, and 12 to 36, respectively. The catastrophic thinking related to pain was assessed using the Brazilian Portuguese Catastrophizing Scale (BP-PCS; Sehn et al., 2012). To measure the pain intensity during the most part of time in the last week was used the VAS, ranging from 0 cm (no pain) to 10 cm (worst possible pain). We used a standardized questionnaire to assess demographic data and medical comorbidities.

As subjects with chronic pain usually use rescue analgesics changes from week to week according to pain level, the analgesic use was defined as the self-reported average used per week during the last 3 months. For data analysis, we included the analgesic use as a dichotomous variable: the analgesic was coded one when they used more than 4 days per week while the analgesic uses less than 4 days per week it was coded as zero (reference value).

### Outcomes

The primary outcomes were the MEP and ICF as assessed by TMS. The secondary outcomes were the cortical excitability parameters CSP and SICI, serum BDNF level, HPT, and the disability related to pain assessed by Brazilian Profile of Chronic Pain: Screen score (B-PCP:S). The primary factor of interest, the score on NPS (0–10) during the conditioned pain modulated (CPM-task), are described in detail below.

- The Brazilian Profile of Chronic Pain: Screen (B-PCP:S; Caumo et al., 2013) was used for quick identification of an individual's multidimensional pain experience. The B-PCP:S includes a severity scale (four items; possible score range of 0–32), an interference scale (six items; possible score range of 0–36), and an emotional burden scale (five items; possible score range of 0–25). The disability related to pain (DRP) regarding severity, interference with daily activities, and the emotional burden was evaluated using the B-PCP:S (Caumo et al., 2013). It accepted as a criterion to define disability a presence of chronic or recurrent pain or discomfort causing restriction (Caumo et al., 2013); thus, we assumed that higher scores on the B-PCP:S indicated more severe disability or greater functional deficits at work, at home, and during social situations and a higher emotional burden (Vidor et al., 2014).
- To measure the cortical excitability parameters we used a surface electromyography. The recordings were gathered at the contralateral right first dorsal interosseous muscles using Ag/AgCl electrodes. First, the resting motor threshold (RMT) was determined by obtaining five motor evoked potentials (MEPs) with a peak-to-peak amplitude of 50 μV from 10 consecutive trials. To define the MEP we recorded 10 MEPs with an intensity of 130% of the individual RMT. Moreover, the cortical silent periods (CSPs) were assessed during muscle activity by a dynamometer to maintain them at ~20% maximal force. Accordingly, the CSPs were 10 records using an intensity of 130% of the RMT. Short intracortical inhibition (SICI) using an inter-stimulus interval of 2 ms was also assessed. The conditioning stimulus was set at 80% of the RMT while the test stimulus was set at 100% of the individual MEP intensity. The intracortical facilitation (ICF) was assessed with an inter-stimulus interval of 12 ms. We conducted the paired-pulse in a randomized order for a total of 30 trials (ten each for ICF, SICI and control stimuli). To calculate the RMT we used the lowest stimulus intensity that was able to evoke an MEP of at least 50 μV in 5 out of 10 consecutive trials. Off-line analyzes included the collection of the duration of the CSPs as well as the amplitudes of all of the MEPs, SICIs, and ICFs. The corresponding units for these parameters included MEP in μV, SICI, and ICF in their ratios to MEP and CSP in ms (Pascual-Leone et al., 1994).
- The laboratory outcome measured was the serum level of BDNF. We collected the blood samples before starting the assessment. We centrifugate the blood samples for 10 min at 4500 × g at 4°C, and we stored the serum at −80° C for the hormone assay. We determined the serum BDNF using an Enzyme-Linked Immunosorbent Assay (ELISA) using a ChemiKine BDNF Sandwich ELISA Kit, CYT306 (Chemicon/Millipore, Billerica, MA, USA). The lower detection limit of the kit for BDNF is 7.8 pg/mL.
- We used the Quantitative Sensory Testing (QST) to assess HPT. This measure use the method of limits with a computer Peltier-based device thermode (30 × 30 mm; Schestatsky et al., 2011) attached to the skin on the ventral aspect of the mid-forearm. The set at 32°C and was increased at a rate of 1°C/s to a maximum of 52°C. The heat pain threshold (HPT) of each patient was defined as the mean of three assessments performed with an inter-stimuli interval of 40 s (Schestatsky et al., 2011). The thermode position was slightly altered between trials, to avoid, either sensitization or response suppression of the cutaneous heat nociceptors.
- To measure the CPM-task we evaluated the pain intensity in two tonics HPT test stimuli separated by a CPM-task. We used the HPT as conditioning pain stimulus to elicit a prolonged pain sensation to trigger CPM. The CPM-task consisted of immersion of non-dominant hand in cold water at a temperature of 0−1°C for 1 min. To maintain the water temperature zero to 1°C was used a thermostat to control the temperature variation. The QST procedure was introduced after 30 s of cold-water immersion. To determine the CPM we used the difference between the pain score on NPS (0–10) QST during cold water immersion (QST+CPM) at the temperature of the point at which subjects felt 6/10 pain on the NPS scale [during the initial time period (T0)]. An accepted criterion to define responders to the CPM-task is the reduction of NPS pain scores under a heterotopic stimulus compared with NPS pain scores under a nociceptive stimulus without a heterotopic stimulus. If the patients did not report a reduction or report an increase in their pain score during the CPM-task, the descendent modulatory systems were considered to have failed to modulate the nociceptive response. For the data analysis, non-responders showed a difference in the score on NPS, HPT1–HPT0, of zero or higher, and for responders, these values were lower than zero.

### Statistical analysis

Descriptive statistics were used to summarize the main socio-demographic features of the sample. *T*-Tests for independent samples and Chi-squared and Fisher's exact tests were used to compare continuous and categorical variables between groups respectively. To test for normality was used the Shapiro-Wilk test. To ensure that the data were normally distributed, we performed a log transformation for BDNF level.

After verifying the corresponding assumptions, the Pearson correlation coefficient (*r*) was used to assess the relationship between covariates (age, sleep quality, catastrophic thinking about pain; state-trait anxiety, and depressive symptoms) with the outcomes related to cortical excitability parameters, BDNF, and pain measures (see **Table 3**). To maintain the assumption of independence between covariates and to control for collinearity when the Pearson correlation coefficients (*r*) for two variables were higher than 0.5 (moderate), in the multivariate analysis model was included only one of the variables (see **Table 4**). Based on this criterion the catastrophizing thinking related to pain and trait-anxiety were included in the multivariate analysis model, taking into account that they have been shown to be correlated with cortical excitability in previous studies on MPS (Volz et al., 2013b; Vidor et al., 2014) (**Table 4**). The covariates not included in the multivariate analysis model were age, depressive symptoms, sleep quality, and state-anxiety. A multivariate covariance analysis (MANCOVA) model was used to explore the relationship between the responders and non-responders to multiple outcomes [cortical excitability (MEP, ICI, ICF, CSP), BDNF, HPT, and disability related to pain on B-PCP:S. Bonferroni's Multiple Comparison Test was used to identify the source of significant differences. The data were analyzed using SPSS software version 22.0 (SPSS, Chicago, IL).

## Results

### Patient characteristics

We screened 54 potential participants with a diagnosis of MPS, and we included 33 in the study. The reasons for exclusion were not fulfilling the diagnostic criteria for MPS, not present a neuropathic component according to the DN4 (Neuropathic Pain Diagnostic Questionnaire), lacking disability as defined in the protocol, and the presence of another diagnosis (fibromyalgia). All enrolled subjects participated in all aspects of the study and were included in all of analyses (Table 1).

**Table 1:** **Demographic and clinical characteristics of the study sample**.

**Variables**	**Non responders (*n* = 23)**	**Responders (*n* = 10)**	***P***
Age (years)	43.36 (14.78)	48.30 (9.13)	0.33
Marital status (married/unmarried)	13/10	4/6	0.31
Education (years)	13.91 (4.25)	12.57 (3.88)	0.37
Smoking (yes/no)	1/22	0/10	0.69
Alcohol consumption (yes/no)	22/1	10/0	0.69
Duration of pain (years)	6.04 (1.64)	6.4 (0.97)	0.42
Pain on visual analog scale (cm)	8 (1.33)	6.5 (1.85)	0.01
Trait-anxiety (STAI-T)	28.82 (6.14)	25.17 (6.41)	0.13
State-anxiety (STA-T)	30.0 (8.42)	29.91 (5.75)	0.98
Beck depression inventory	13.45 (7.04)	15.83 (7.12)	0.37
Brazilian Portuguese Catastrophizing Scale (BP-PCS)	33.82 (6.98)	31.52 (8.14)	0.42
Number of days analgesics were used per week in the last 3 months (< 4 times/= 4 times)^a^	8/15	2/8	0.33
Presence of other chronic diseases before appearance of pain (yes/no)^b^	4/19	2/8	0.6
Diagnosis of psychiatric disorders (yes/no)	8/15	5/5	0.32
Active use of central nervous system medication (yes/no)^c^	20/3	7/3	0.25

*Values are given as the mean (SD) or frequency (n = 33)*.

a *The same patient may have used more than one medication*.

b *Chronic diseases other than pain: hypertension (n = 12); ischemic heart disease (n = 1); heart attack (n = 1); diabetes mellitus (n = 5); thyroid diseases (n = 2); other chronic diseases listed (n = 0)*.

c *Central nervous medication: tricyclic antidepressant (n = 2); topiromate (n = 1); tylex (n = 1)*.

### Univariate analysis

#### Relationships between the function of the corticospinal modulatory system, motor córtex excitability, pain measures, and BDNF level

Relationships between the function of the corticospinal modulatory system, motor córtex excitability, pain measures, and BDNF level according to a spectrum of responders and no responder to CPM-task. The non-adjusted means and standard deviation (SD) of the cortical excitability parameters, BDNF, pain threshold and disability related to pain were presented in Table 2.

**Table 2:** **Measurements of motor córtex parameters by TMS, HPT, B-PCP:S, and BDNF (*n* = 33)**.

**Cortical Excitability Measures**	**Non-responders (*n* = 23)**		**Responders (*n* = 10)**		***P*^&^**
	**Mean ± *SD***	**Median (Q25, Q75)**	**Mean ± *SD***	**Median (Q25, Q75)**	
Motor threshold (MT)	44.46 (8.04)	44 (32: 65)	41.1 (5.53)	40.5 (32; 50)	0.15
Motor evoked potential (mV)	1.93 (0.54)	2.06 (0.98; 3.14)	1.40 (0.27)	1.42 (1.03); 1.81)	0.01
Intracortical facilitation (ratio: ICF/test stimulus)	1.43 (0.3)	1.35 (0.71; 1.99)	1.11 (0.12)	1.09 (0.94; 1.24)	0.00
Short interval intracortical inhibition (ratio: SICI/test stimulus)	0.25 (0.02)	0.25 (0.23;0.27)	0.27 (0.10)	0.25 (0.08; 0.42)	0.38
Cortical silent period (CSP)	69.36 (21.74)	79.00 (38.0;120.0)	61.91 (15.49)	62.50 (33.25; 91.75)	0.17
Profile of chronic pain: screen for Brazilian population (B-PCP:S)	71.00 (10.02)	73.00 (55.0;91. 0)	59.22 (11.23)	63.00 (51.0; 75.0)	0.00
Quantitative sensory testing (°C)	42.78 (4.27)	44 (35;50)	38.0 (3.03)	38.00 (37: 41)	0.00
Brain-derived neurotrophic factor (BDNF) pg/ml (*log*)	32.56 (9.95)	33.0 (20.0;36. 0)	25.59 (10.24)	22.5 (5.5; 39.5)	0.02

*Motor evoked potential: (MEP); Interquartile interval (Q). Intra-cortical inhibition (ICI) expresses the relationship between the amplitude of wave and motor evoked potentials (relative amplitude, express in %), at inter-stimuli intervals (ISIs) of 2 ms with paired-pulse. The first is a sub-threshold stimulus [80% of the rest motor threshold (rMT)] followed by the second one which is a suprathreshold stimulus (130% rMT)*.

(A) Cortical silent period (CSP) expressed in milliseconds (ms);

*(B) Motor-evoked potentials (MEP) expressed in mV, evoked by a stimulus of 130% the intensity of the rMT, and should have peak-to-peak MEP amplitude of at least 1 mV*.

& *, Comparisons of mean using t-test for independent samples*.

#### Assessment of relationship between independent variables to identify potential confounders

The Pearson correlation was used to identify potential confounding factors in the relationships between outcomes (cortical excitability, BDNF, HPT, and disability). The correlated parameters were the scores of the Brazilian Portuguese Catastrophizing Scale (B-PCS); Beck Depression Inventory (BDI); Pittsburgh Sleep Quality Index (PSQI); and Short State-Trait Anxiety Inventory (STAI-E-T), and age (Table 3). The covariates included in the multivariate analysis model (Table 4) were the trait-anxiety and catastrophizing scores.

**Table 3:** **Pearson correlation coefficient (*r*) between potential confounding factors and outcomes (*n* = 33)**.

	**Age**	**STAI-T**	**STAI-E**	**BPC-S**	**B-PCP:S**	**BDI**	**PSQI**	**MEP**	**ICF**	**SICI**	**CSP**	**BDNF**
Age	*r* = *0.05*											
STAI-T	*r* = 001	*r* = *0.15*										
STAI-E	*r* = −0.04	*r* = 0.65^**^	*r* = –*0.07*									
BP-PCS	*r* = 0.06	*r* = 0.32	*r* = 0.29	*r* = –*0.08*								
B-PCP:S	*r* = −0.13	*r* = 0.40^*^	*r* = 0.19	*r* = 0.62^**^	*r* = –*0.11*							
BDI	*r* = 0.18	*r* = 0.58^**^	*r* = 0.43^**^	*r* = 0.66^**^	*r* = 0.54^**^	*r* = –*0.25*						
PSQI	*r* = −0.07	*r* = 0.34^*^	*r* = 0.24	*r* = 0.54^**^	*r* = 0.36^*^	*r* = 0.46^**^	*r* = –*0.11*					
MEP	*r* = −0.26	*r* = 0.05	*r* = −0.08	0.11	*r* = 0.26	*r* = −0.10	*r* = −0.05	*r* = 0.33^*^				
ICF	*r* = −0.01	*r* = 0.15	*r* = −0.06	0.14	*r* = 0.45^**^	*r* = 0.09	*r* = −0.07	*r* = 042^*^	*r* = 0.25			
SICI	*r* = −0.13	*r* = −0.12	*r* = −0.27	–0.01	*r* = −0.03	*r* = −0.03	*r* = −0.06	*r* = −0.15	*r* = 0.04	*r* = −*0.27*		
CSP	*r* = −0.05	*r* = −0.14	*r* = 0.05	–0.18	*r* = −0.13	*r* = −0.13	*r* = −0.21	*r* = 0.01	*r* = 0.27	*r* = 0.18	–0.38^*^	
HPT	*r* = 0.32	*r* = 0.03	*r* = −0.08	0.28	*r* = 0.07	*r* = 0.20	*r* = 0.11	*r* = −0.35^*^	*r* = −0.05	*r* = 0.34^*^	*r* = −0.11	*r* = *0.20*

** *Correlation is significant at the 0.01 level (2-tailed)*.

* *Correlation is significant at the 0.05 level (2-tailed). Brazilian Portuguese Catastrophizing Scale (BP-PCS); Beck Depression Inventory (BDI); Pittsburgh Sleep Quality Index (PSQI); Short State-Trait Anxiety Inventory (STAI-E-T); Brazilian Profile of Chronic Pain: Screen (B-PCP:S); Intra-cortical inhibition (ICI); Cortical silent period (CSP): Motor-evoked potentials (MEP) expressed in mV, Brain-derived neurotrophic factor (BDNF) pg/ml (log)*.

Table 4**Relationship between outcomes (cortical excitability parameters, pain measures and BDNF), and responders and no responders according change in NPS (0–10) during the CPM-task (*n* = 33)**.

**Table:** 

**Dependent variable**	**Type III Sum of Squares**	***df***	**Mean Square**	***F***	***P***	***Partial eta Squared***
Motor evoked potential (mV)	1.15	3	0.38	2.79	0.03	0.22
Intracortical facilitation (ratio: ICF/test stimulus)	2.52	3	0.84	5.81	0.00	0.38
Short intracortical inhibition (ratio: SICI/test stimulus)	0.94	3	0.31	9.19	0.00	0.49
Cortical silent period	0.004	3	0.001	0.17	0.91	0.01
Brazilian profile of chronic pain: screen (B-PCP:S)	929.06	3	309.69	1.10	0.36	0.10
Quantitative sensory testing (°C)	184.73	3	61.58	4.86	0.00	0.33
Brain-derived neurotrophic factor (BDNF) pg/ml (*log*)	2881.96	3	960.65	15.40	0.00	0.61

**Table:** 

**Parameter**	**SEM**	**βa**	***t***	***P***
**MOTOR EVOKED POTENTIAL (mV)**				
Conditioned pain modulation (CPM) during CPM/task				
No responder^a^	0.61	0.15	4.09	0.00^*^
Brazilian Portuguese catastrophizing scale (BP-PCS)	0.007	0.009	0.74	0.46
State-anxiety (STAI-T)	−0.01	0.01	−1.50	0.14
**INTRACORTICAL FACILITATION (RATIO: ICF/TEST STIMULUS)**				
Conditioned pain modulation (CPM) during CPM/task	0.33	0.07	4.50	0.00^*^
No responder^a^				
Brazilian Portuguese catastrophizing scale (BP-PCS)	0.004	0.004	0.88	0.38
State-anxiety (STAI-T)	0.004	0.006	0.70	0.48
**SHORT INTRACORTICAL INHIBITION (RATIO: SICI/TEST STIMULUS)**				
Conditioned pain modulation (CPM) during CPM/task	−0.02	0.03	−0.62	0.54
No responder ^a^				
Brazilian Portuguese catastrophizing scale (BP-PCS)	−0.01	0.02	−0.34	0.80
State-anxiety (STAI-T)	1.85	0.003	0.007	0.99
**CORTICAL SILENT PERIOD**				
Conditioned pain modulation (CPM) during CPM/task	10.82	6.66	1.62	0.11
No responder^a^				
Brazilian Portuguese catastrophizing scale (BP-PCS)	−0.19	0.40	−0.47	0.64
State-anxiety (STAI-T)	−0.47	0.50	−0.94	0.36
**BRAZILIAN PROFILE OF CHRONIC PAIN: SCREEN (B-PCP:S)**				
Conditioned pain modulation (CPM) during CPM/task	7.81	3.13	2.48	0.01^*^
No responder^a^				
Brazilian Portuguese catastrophizing scale (BP-PCS)	0.80	0.18	4.24	0.00^*^
State-anxiety (STAI-T)	0.51	0.24	2.17	0.03^*^
**QUANTITATIVE SENSORY TESTING (°C)**				
Conditioned pain modulation (CPM) during CPM/task	−3.82	1.41	−2.70	0.01^*^
No responder^a^				
Brazilian Portuguese catastrophizing scale (BP-PCS)	0.08	0.08	0.99	0.33
State-anxiety (STAI-T)	−0.19	0.10	−1.82	0.07
**BRAIN-DERIVED NEUROTROPHIC FACTOR (BDNF) PG/ML (*****log*****)**				
Conditioned pain modulation (CPM) during CPM/task	0.39	0.14	2.66	0.01^*^
No responder^a^				
Brazilian Portuguese catastrophizing scale (BP-PCS)	−0.05	0.09	−0.61	0.54
State-anxiety (STAI-T)	−0.01	0.01	−1.47	0.15

a *Reference category is no responder, hence a positive value mean that the mean was higher in no responder*.

CPM-task [no responder (NPS (0–10) HPT1–HPT0 ≥ 0) or responder (NPS (0–10) HPT1–HPT0 < 0]

* *P < 0.05*.

### Multivariate analysis of the relationship between the corticospinal modulatory system, cortical excitability, BDNF, HPT, and disability according to spectrum of responders and non-responders to CPM-task

The results of the MANCOVA model analysis with multiple outcomes as dependent variables, including cortical excitability parameters (MEP, ICF, SICI, CSP), BDNF, HPT, and disability related to pain according to spectrum of responders and non-responders to CPM-task, and the STAI-E-T score and catastrophizing score, as independent variables, are presented in Table 4. The MANCOVA model using Bonferroni's Multiple Comparison Test revealed a significant relationship between the responders and non-responders groups and the outcomes related to cortical excitability measurements (ICF and MEP), BDNF, disability related to pain and HPT [Hotelling's Trace = 1.84, F(34) = 6.05, *P* < 0.001]. This analysis presented a power of 0.99. The adjusted determination coefficient of this model was R2 = 0.57; thus, the variables included in the model explain 57% of the variance in the outcome variables. The results of this adjusted multivariate model are presented in Table 4. Non-responders showed higher cortical excitability (ICF, MEP), greater disability related to pain, higher BDNF level, and lower HPT. However, no effect was observed in other cortical excitability parameters (CSP and ICI; see Table 4).

**Figure 2:**
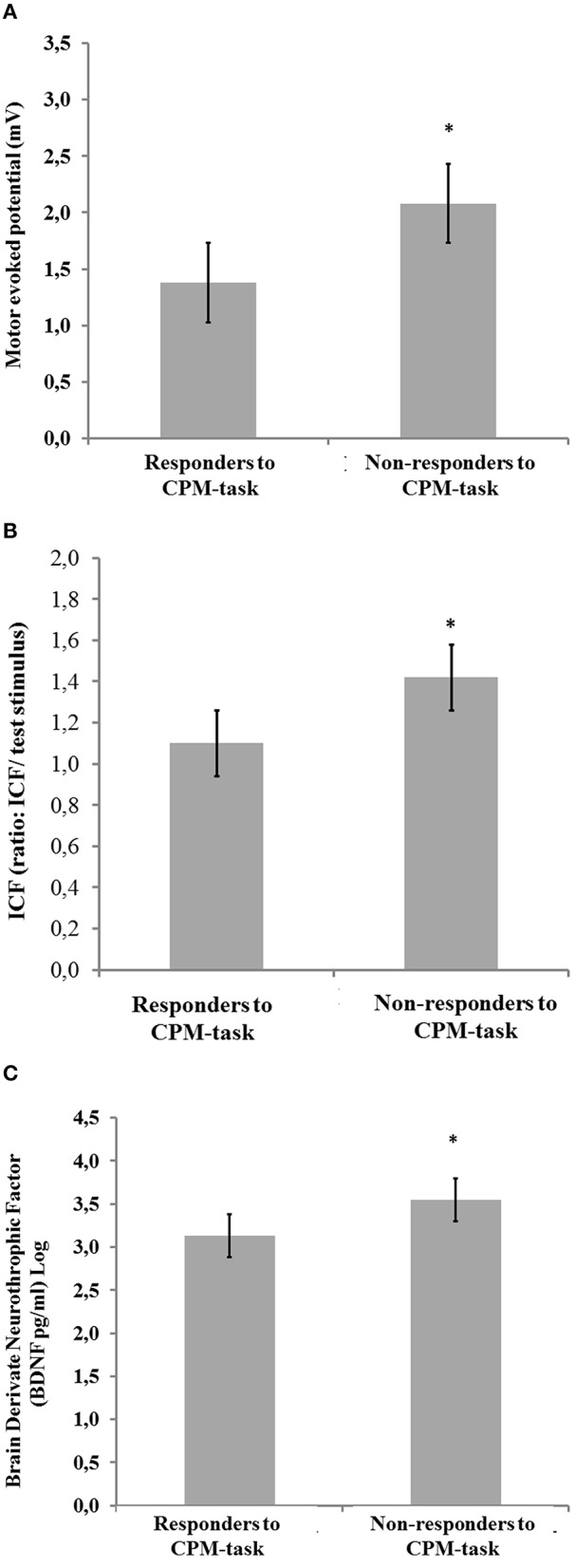
**Comparisons between [non-responders (NPS (0–10) HPT1–HPT0 ≥ 0; *n* = 10) and responders (NPS (0–10) HPT1–HPT0 < 0; *n* = 23)]**. **(A)** Motor evoked potential (mV); **(B)** Intra-cortical facilitation (amplitude/MEP amplitude ratio = ICF); and **(C)** Brain derived neurotrophic factor (BDNF) ng/ml (Log). Error bars indicate standard error of the mean (S.E.M.). Asterisks positioned above the bars indicate differences between groups (responders and non-responders to CPM-task) assessed by MANCOVA with *post-hoc* Bonferroni's Multiple Comparison test.

In Figures 2A–C are presented the relationships according to a spectrum of responders and non-responders to CPM-task and intracortical facilitation and MEP (primary outcomes) and BDNF (secondary outcome). The means were compared using MANCOVA with Bonferroni's Multiple Comparison test (the model was shown in Figures 2A–C; Table 4).

## Discussion

This study confirmed our hypothesis that the descending pain modulation system as assessed according to a spectrum of responders and non-responders to CPM-task is simultaneously correlated with a disinhibition at the cortical level, as measured by ICF and with global neuroplasticity levels as determined by serum BDNF. Also, the disengagement of descending pain modulatory system was correlated with a dysfunction of the corticospinal pathway as indexed by MEP, a lower HPT, and a greater disability.

The current study expanded on the data available in the literature showing that the magnitude of disinhibition in regulating sensory information was associated with changes in the cortical and subcortical levels. This disinhibition state occurs through multiple neurobiological systems, which can amplify sensory pain signals to the neural pain matrix. Additionally, the level disengagement of descending pain modulatory system was correlated with changes in serum BDNF level, which is involved in the modulation of the excitatory/inhibitory central nervous system balance. Thus, the variation in the spectrum of dysfunction of internal modulator system in chronic pain conditions could be understood as a signal from a balance in the neuroplasticity mediators involved in the modulation of the excitatory/inhibitory central nervous system (CNS; Deitos et al., 2015). While that the variation of BDNF could be interpreted as a signal from a “diseased balance,” once such balance differs between the spectrum of responders and responders to CPM-task. However, persists the concerns how good is this signal to identify the chronic pain imbalance in the CNS and how is its predictive properties for the evaluation of the MPS.

These results demonstrated that this integrative pattern to assess changes in the pain pathway highlights that a cross talk between the neural network of cortical regions and the spine-bulbospinal loop occurs along with changes in the BDNF secretion, which is the central marker of neuroplasticity process mechanisms. Thus, this set of changes reinforcing the hypothesis, that, if we improve the understanding of underlying neurophysiological mechanisms of chronic MPS, this could give support for the clinical decision based on practical approaches for its recognition (Nijs et al., 2010). Additionally, these findings provide some theoretical support for the mechanism involved in the effect of interventions that improved pain and enhanced the function of the descendent modulatory system in studies that used melatonin, amitriptyline (de Zanette et al., 2014), rTMS (Dall'Agnol et al., 2014), and the combination treatment of CPM and duloxetine (Yarnitsky et al., 2012). Although human studies permit us to determine only the effect in the network, our findings allow a new way to construct the rational to combine therapeutic approaches to improve functional of descending pain modulatory systems. Such techniques include pharmacological (i.e., antidepressant, anticonvulsant, etc.) and non-pharmacological approaches (i.e., Transcranial direct current stimulation (tDCS), TMS, electroacupuncture and other physical therapy).

We observed greater MEPs amplitude in non-responders to CPM-task (Table 4). Although a significant portion of the corticospinal input to the motoneuron pool is relay via lumbar group II interneurons (Marchand-Pauvert et al., 1999), the MEP amplitude is a reflection of the latency of depolarization of the spinal motor neuron pool. Its amplitude reflects the integrity and function of conduction along the efferent pathway, which form part of the lumbar propriospinal system and it express the excitability of the cortical and spinal motor neuron pool (Marchand-Pauvert et al., 1999; Pierrot-Deseilligny and Burke, 2005; Iglesias et al., 2008). Thereby, this result suggests that an enhanced activity of descending tracts, whose motor portion is assessed by the MEP, suggests that the inhibitory capacity of the corticospinal modulator system is reduced (Vidor et al., 2014), resulting in increased amplitude of MEP. One critical issue here is whether corticospinal excitability is a compensatory or a causal mechanism of pain. Given our data does not allow us to clarify the temporal relationship between these two variables; we can only hypothesize the correlation between these two variables (MEP and CPM response). We have proposed before that increased motor cortex excitability is a compensatory mechanism aiming to reduce thalamic overactivity and thus pain (Castillo Saavedra et al., 2014); though this mechanism is not enough to control pain (an anology here would be increased insulin in a subject with hyperglycemia; increased insulin levels would be the compensatory mechanism). Therefore, increased pain increases corticospinal excitability and when pain is controlled this marker becomes normalized. The data from ICF supports this hypothesis. We have proposed before that increased motor cortex excitability is a compensatory mechanism aiming to reduce thalamic overactivity and thus pain (Castillo Saavedra et al., 2014).

Either increased ICF or decreased ICI suggest an involvement of cortical mechanisms in the dysfunction of the descendent modulatory system, which facilitate the activity of the corticospinal system. Although the ICF is a complex phenomenon, it reflects increase in the activity within glutamatergic circuits, it also may arise through a loss of GABA-A-mediated modulation (Di Lazzaro et al., 2000; Fedi et al., 2008). Additionally, the disinhibition involves the loss of inhibitory pyramidal cells. MEP amplitude is also an indicator of primary motor córtex excitability: larger amplitudes indicate higher excitability of the motor córtex, which may modulate intracortical excitability and the transmission efficiency of corticospinal neurons, resulting in less facilitation. Overall, as proposed above these changes in cortical plasticity could be explained as a compensatory mechanism to downregulate increased excitability in the pain neural networks such as thalamic structures.

The higher serum BDNF in non-responders suggests that this neurotrophin may be a marker of severity of CS. The CS involves a proliferation of synaptic activity due the trophic factors, to support maladaptive plasticity that perpetuates the sensation of pain. Our findings give neurophysiological support (MEP) to understand the link between serum BDNF and the severity of dysfunction of the descendent modulatory system. Even though this relationship is complex, they support the idea that the activity of the descending inhibitory system is related to central sensitization (Schwenkreis et al., 2003; Deitos et al., 2015) and a greater activation in the brainstem (Graven-Nielsen et al., 2012). This assumption, supported by an experimental study with rats exposed to chronic pain, demonstrates that the BDNF effect on pain pathways may change according to the region of central nervous system (i.e., spine, brainstem, hippocampus, and cortex, etc.; Spezia Adachi et al., 2015). The mentioned study demonstrated that the tDCS decreased the BDNF levels in the spinal cord and brainstem, whereas BDNF levels did not change in the hippocampus (Spezia Adachi et al., 2015). These differents effects according to site suggest that BDNF activates distinct pathways (i.e., descending systems) and that its effect is pleiotropic. Although previous findings show that the increase in excitatory activity and the decrease in inhibitory synaptic activity in the córtex related to BNDF level (Ren and Dubner, 2007; Tao et al., 2014), the present results do not allow for a conclusion regarding a cause-effect relationship between BDNF level and descendent modulatory system dysfunction.

Overall, the findings of this study corroborate the idea that the BDNF modulates the synaptic plasticity in an activity-dependent manner to strengthen a nociceptive transmission, recruits non-nociceptive input to the pain pathways and it binds to high-affinity trkB receptors. This BDNF effect enhances the response that NMDA-mediated C-fibers evoke, which in turn causes activation of several signaling pathways in spinothalamic tract neurons. Thereby, this strength excitatory synapsis promotes the disinhibition of descending pathways (Zanette et al., 2014). This statement is also supported indirectly by clinical findings, where the serum BDNF was correlated inversely with the pressure pain threshold in fibromyalgia (Zanette et al., 2014). Equally, we showed that the BDNF increase would be favoring pain transmission because greater scores in the CPM indicates a lower function in the descending pain modulatory system and a higher propensity for pain. This finding is biologically plausible because the enhance in the BDNF activates signaling pathways in the spinothalamic tract, which reduces the GABAergic inhibitory effect (Spezia Adachi et al., 2015). These findings support the hypothesis that the chronic pain induces reorganization in circuits involved in pain processing at cortical and in descending pain modulatory system. Although the relationship between BDNF with the physiopathology of pain is complex, it has important functions in the processes of neurogenesis and neuroplasticity. Thereby, efforts are being made to understand its role in the pain modulatory system.

In the current study, a lower HPT and higher DRP in non-responders were observed (Table 4). These results are congruent with evidence from previous studies that a lower pain threshold in patients with long-term chronic pain may be a signal of lack of function of the inhibitory system (Kwon et al., 2013; Defrin et al., 2015). Another explanation for this finding is a potential protective effect if one considers that the hippocampus amplifies signals to the neural representation (Ma et al., 2012).

A greater disability according to scores on the B-PCP:S was associated with the disinhibition of the descendent pain modulatory system. The B-PCP:S dominions indicate pain severity, restriction for daily activities (at work, at home, during social situations) and the emotional burden. According to a spectrum of responder and non-responders to CPM-task, the disability was correlated positively with the catastrophizing and trait-anxiety. In previous study we demonstrated the relationship between greater disability related to pain and a higher trait anxiety in MPS (Vidor et al., 2014). While in another study with healthy subjects was observed that the perceived intensity of the conditioning stimulus was associated with the pain catastrophizing and trait anxiety (King, 2014). In fact, the current findings suggest that the relationship between the descending modulatory system and the disability related to pain is regulated by brain regions that are involved not only with pain but also with cognitive and emotional functioning in general (Pessoa, 2008). Similar dysfunction was observed when there were lesions in brain regions implicated in descending pain modulation (i.e., traumatic brain injury and multiple sclerosis), including the medial prefrontal córtex (PFC) and rostral anterior cingulate córtex (ACC; Bushnell et al., 2013). Additionally, it has been demonstrated that the alterations in the biological integrity and functioning of brain regions were involved in both pain control and cognitive and emotional functioning. Thereby, the changes in this network could explain the relationship between the severity disability and the dysfunction of the corticospinal pain modulatory system (Table 4).

This study had some limitations: Firstly, TMS is an indirect neurophysiological evaluation of neurotransmitter system activity. Secondly, only females were evaluated, as gender differences in pain perception and modulation are controversial. Thirdly, psychiatric disorders are a potential confounding factor in chronic pain syndromes, and they cannot have been adequately controlled. The psychiatric symptoms (anxiety, depression, catastrophizing, and psychiatric diagnosis) were equally distributed between the groups (responder vs. non-responder). In fact, 39.39% (14/33) of patients suffered from mental illnesses. However, this finding is expected, because the emotional disturbance is part of chronic pain syndromes, and they can worsen the sensitization and chronification. Moreover, the results of this study need to be carefully interpreted because it was an exploratory study. Further, research on chronic pain of different psychopathologies is required to confirm our initial findings and the impact of our findings on patients' responses to different therapeutic approaches.

These results suggest that a non-response to the CPM-task is likely due to increased plasticity to in central structures associated with pain that control endogenous inhibitory control and that in this case compensatory mechanisms are activated as reflected by increased cortical excitability. This failure to respond to CPM-task is associated with higher serum BDNF, lower HPT, and a greater level of disability related to pain. Overall, these findings suggest that the CPM-task is a test that allows for inference regarding the loss of net descending pain inhibition. Thus, this short and simple test might be useful for predicting a patient's response to therapy, and it helps in the clinical decision-making process for individual patients. The results of this study may also assist in the development of individualized treatment.

## Author contributions

AB participated in the sequence alignment and drafted the manuscript. MT participated in the sequence alignment. ES participated in the design of the study and performed the statistical analysis. FG conceived the study, participated in its design and coordination and helped drafting the manuscript.

### Conflict of interest statement

The authors declare that the research was conducted in the absence of any commercial or financial relationships that could be construed as a potential conflict of interest.
